# Quantifying people’s experience during flood events with implications for hazard risk communication

**DOI:** 10.1371/journal.pone.0244801

**Published:** 2021-01-07

**Authors:** Nataliya Tkachenko, Rob Procter, Stephen Jarvis

**Affiliations:** 1 Smith School of Enterprise and the Environment, School of Geography and the Environment, Oxford University Centre for the Environment, University of Oxford, Oxford, United Kingdom; 2 The Alan Turing Institute, The British Library, London, United Kingdom; 3 Department of Computer Science, University of Warwick, Coventry, United Kingdom; 4 College of Engineering and Physical Sciences, University of Birmingham, Birmingham, United Kingdom; Beihang University, CHINA

## Abstract

Semantic drift is a well-known concept in distributional semantics, which is used to demonstrate gradual, long-term changes in meanings and sentiments of words and is largely detectable by studying the composition of large corpora. In our previous work, which used ontological relationships between words and phrases, we established that certain kinds of semantic *micro-changes* can be found in social media emerging around natural hazard events, such as floods. Our previous results confirmed that semantic drift in social media can be used to for early detection of floods and to increase the volume of ‘useful’ geo-referenced data for event monitoring. In this work we use deep learning in order to determine whether images associated with ‘semantically drifted’ social media tags reflect changes in crowd navigation strategies during floods. Our results show that alternative tags can be used to differentiate *naïve* and *experienced* crowds witnessing flooding of various degrees of severity.

## Introduction

Unusual events and changes in the natural environment can significantly impact people’s day-to-day activities, therefore information on human mobility has been primarily valued for its crucial role in response to disaster and evacuation strategies [1]. Some studies have reported that the success of planning and executing evacuation operations to a great extent depend on exact information of where people are [2, 3]; other studies mention that real-time designation of the risk areas could benefit from the human movement patterns [4]. Also, successful geo-targeting of appropriate shelter locations relies on ‘hot-spots’, that is vulnerable gatherings of people [5, 6], whereas adaptation of early and real-time warning communication to mobile outdoor populations can be instrumental for the deployment of a new generation of *smart alert systems* [7, 8].

Despite its obvious importance, studies of human mobility during natural disasters (i.e., under conditions of disruption) are quite scarce. [9, 10]. The majority of studies seem to primarily concentrate on the fundamental characteristics of *generic* human mobility patterns [1], which fall into categories of *small world behaviours* [11] presuming the existence of *cliques* and generally predictable activities, *Lévy Flights* of the exploratory chaotic movements or *Brownian navigation* associated with aggressive, proactive or predatory motives [12–19].

Researchers at Harvard University [1] looked at 2-year human mobility data collected from Twitter for a number of different natural disasters around the world, including hurricanes, winter storms, wildfires, rainstorms and earthquakes, in order to understand whether major events can significantly perturb routine mobility patterns described by *power law* distributions. They introduced the concept of the *quantitative resilience* of human mobility, according to which it is possible to evaluate the degrees of interdependence between people’s spatial movements and civil infrastructure, such that resilient activity is able to return promptly to its steady state equilibrium in response to natural hazards. They concluded that although perturbed by various hazards, the movement of people in almost all cases studied still conformed to a natural-state power law distribution, whereas event characteristics, such as *severity* and *duration*, tended to lead to much more significant disruption of urban mobility under natural hazard conditions.

While spatio-temporal data signals are useful for crowd estimation and intervention planning, research on human sensory experience during natural events is, to the best of our knowledge, nonexistent. However, as social media is rapidly becoming more *visual* and less *textual* [20, 21], the need also increases to adapt meaning extraction strategies from various *sensory* data modalities (e.g., video, audio, images). Specifically, for natural hazard analytics such data transformations hold a lot of promise, since it is widely known that in situations of uncertainty people tend to generate a lot of mediated information when exploring their environment and adapting to it [22]. In this study we therefore propose to extend existing work on human *mobility resilience* as an impact indicator of natural disaster to *spatial navigation strategies*, which can not only quantify, but also describe crowds exposed to conditions of great uncertainty during an evolving hazard situation. Building on previous findings, we focus on one natural hazard event only (flooding), however, this time accounting for the attribute of *severity* [1].

This study is based on geo-referenced (XY) information from the Yahoo! Flickr platform, where the data consists of images associated with descriptive text. Falling into the time period of 2004-2014, only data geographically associated with UK floodplain areas used for 3-stage risk communication (‘Alerts’, ‘Warnings’ and ‘Severe warnings’) was extracted. Selected data entries were then filtered to extract the following three categories: **(a)** direct event descriptors (i.e., tags, containing word ‘flood’, **(b)** benchmark lexemes (i.e., ones with which semantically unstable words highly correlate, such as ‘nature’ and ‘landscape’, and **(c)** alternative (i.e., semantically unstable) lexemes ‘river’ and ‘water’. This framework was derived from our previous findings on the positive role of ‘semantically drifted’ material in flood event monitoring [23]. By analysing and comparing data across the 3-stage severity codes and before/after they have been issued, we attempt to understand how navigational strategies of crowds can be used for event segmentation according to their sensory experiences of the hazard.

The paper is organised as follows. After reviewing related work about spatial navigational strategies, we propose three hypotheses. This section is then followed by a description of the data collection and analytical methods. Then we present results, findings and their implications for the wider field of event analytics. Finally, we present our conclusions and assess the study’s limitations.

## Materials and methods

Spatial navigation belongs to those fundamental behaviours that are essential for everyday life. It serves the purpose of survival and involves nearly all sensory systems, though visual information appears to prevail while traversing the environment in a purposeful manner [24–26]. Also, as a research branch of behavioural geography it is primarily concerned with the question of how spatial information such as *orientation (or direction)* and *attention (or focus)* are coded cognitively. Specifically, whether this is done *egocentrically* (i.e., in direct relation to the observer as a primary reference point) or *allocentrically* (when the reference is a visual frame situated independently from the observer’s position in space). The question of what is the difference between the strategies from the perspective of their cognitive underpinnings remains a topic of debate [27].

### Spatial focus

One of the ways to approach this difference empirically is to understand how people relate to the components of their surrounding environments. That is, whether they treat them as generic *objects* or distinguish as the *landmarks* [28, 29]. There are currently many outstanding questions about the roles of ‘landmarks’ and ‘objects’ in guiding human behaviour, however, the primary difference between them lies in the fact that ‘landmarks’ are used for orientational purposes, while ‘objects’ merely contribute to the contextual background and accrue various associative properties. It has also been argued that specialisation of ‘objects’ as ‘landmarks’ should be based on the *function* of the ‘object’ within a specific navigational context. Where appearance is concerned, the more distinctive an ‘object’ looks within an environment (and more informative or memorable its features are), the more likely it is associated with the ‘landmark’ category [30]. Also, the *stability* of ‘objects’ in the environment can influence their role as ‘landmarks’. If the former are to be counted as ‘landmarks’, then they need to be able to provide reliable navigational information, predominantly at the expense of a stable spatial position as it has been previously shown that objects at decision points are better remembered than those at non-decision locations [31, 32]. A study of virtual route-navigation by [33] demonstrated how objects in the environment attain action-related associations. And although ‘landmarks’ are commonly referred to as discrete objects, the geometry of their extended surface or boundaries can also provide important information for navigation.

### Spatial orientation

It is already known that environmental orientation is a crucial component of successful spatial navigation. During navigation, a sense of direction can help us to establish an understanding about spatial relationships between different locations and can improve the representational stability of situated real-world objects [34].

For humans, orientation and directional information are controlled predominantly by visual cues and hence it can be argued that for successful navigation in space one needs to operationalise already accumulated storage of visual information about previously visited locations or to create new mental images for current or future references. Performance for aligned versus misaligned (or *connected* vs. *disconnected*) orientations is therefore considered to reflect the fact that semantic relationships between objects in the real world are assigned similar connections in memory with respect to the specified reference direction.

### Environmental spaces

Relationships between space and people’s experiences have been well covered in Ittelson’s (1973) theory [35], where he draws a distinction between the ‘space of objects’ and what he termed as ‘environmental spaces’. Unlike *spaces of objects*, which are usually smaller than the human body, *environments* necessitate movement within them in order to be perceived and experienced. Furthermore, unlike *object spaces*, which have little emotional content, environmental spaces also foster affective attachments, thus influencing perception of the environmental space as a whole.

Similar to this framework, behavioural geography introduced in the 1990s a distinction between *perceptual* and *cognitive* spaces. According to this, perceptual spaces refer to what can be seen or observed through the senses at one time, whilst the cognitive ones comprise larger-scale spaces, which cannot be sensed at once by our sensory system and therefore must be consecutively assembled, much like a jig-saw puzzle [36]. Cognitive spaces are also considered instrumental in linking sensory images of our immediate experiences to cognitive factors relating to beliefs, knowledge and memory.

Since different parts of the environment are represented independently, for successful navigation these independent representations have to be linked. Graph-like representations have long been suggested as suitable a structure to integrate these spatially independent, yet semantically interconnected, experiences or memories of space [37–39].

In these graph-like structures, local positional information is usually attached to nodes, while edges are used to reflect the strength of the connections between them. The exact nature of information stored in nodes and edges can differ between models. Thus [38], for example, suggested that nodes are place representations, while connections between distinct places are encoded as vectors in the polar coordinates of a two-dimensional coordinate system in which each point is determined by a distance from a reference point and an angle from a reference direction. Closely related to the Poucet’s *network of charts* is the *theory of the network of reference frames* [40], which suggests that environmental spaces are represented by means of interconnected reference frames, i.e., independent coordinate systems, each with its own specific orientation. Nevertheless, irrespective of structural differences, the importance of these theories lies in their efforts to structure our everyday mobility strategies according to network theory.

### ‘Wayfinding’ and ‘route-following’ navigation

Functional brain imaging studies in people [41] have demonstrated that the hippocampal circuit is recruited when people employ strategies and require allocentric processing, such as planning new routes through unfamiliar spaces or tracing novel ones through familiar environments (i.e., ‘wayfinding’ navigation). The parietal cortex and striatal circuits, in contrast, are involved in egocentric navigation strategies, such as following already known trajectories (i.e., ‘route-following’ strategy). These findings instigated some further adaptations of network theories to the more recent concepts of *focus* and *orientation* mentioned above.

During ‘wayfinding’, landmarks specifying focal location are usually rare or altogether absent during the navigation course, while ‘route-following’ can be characterised by well defined areas of concentration throughout the entire trajectory due to route familiarity.

Apart from attention strategies, navigation in environmental spaces also requires knowledge about connections between places, similar to the graph-like representations, where nodes represent places and edges, i.e., navigational connections-relationships between scenery objects or landmarks. During ‘wayfinding’ such connections are usually absent, whilst ‘route-following’ is characterised by semantically connected objects or landmarks.

Since dedicated data design and collection for such a study can be very costly, we turned our attention to freely available social media information containing semantically tagged photographs, associated with point (XY) geo-location metadata (Yahoo! Flickr platform). Specific interest in the visual modality is motivated by [42], who discovered that scene statistics generated by a classification algorithm can categorise scenes in the way humans do. For example, people tend to classify natural scenes according to the co-occurrence of objects (i.e., ‘water’, ‘sun’ and ‘sunbathers’ would mean for an observer that she is looking at the beach; and in reverse, the natural scene ‘beach’ can be used to elicit the recall of all the above-mentioned objects). These findings are also claimed to be supported by earlier studies [43, 44] on the *speed* of identification of contextually consistent objects.

In this study we will rely on deep learning algorithms in order to extract objects from images of scenes, which can be subsequently used to classify navigational strategies of flood eyewitnesses.

### Hypotheses

Analysis of navigational strategies during flood events can be sensitive to both spatial and temporal design constraints. For example, we can characterise public behaviour during individual events for a particular area across multiple events or across events of a similar degree of severity. As these first two questions would form a nice exploratory analyses for subsequent case studies, we decided to follow this route, with the aim of understanding public behavioural response to flood warning information [45–47].

In order to evaluate human navigational behaviour across floodplains during each of 3 levels of flood risk notification (i.e., ‘Alerts’, ‘Warnings’ and ‘Severe warnings’), we selected images from the Yahoo! Flickr platform that are tagged with either of the three groups of keywords: (a) direct event descriptors (i.e., ‘flood’); (b) *alternative* lexemes exhibiting transient semantic drift around flood events (‘river’, ‘water’) [23] and *benchmark* lexemes used to describe the general, undisturbed state of the natural landscape of floodplains (i.e., ‘nature’, ‘landscape’).

Since no study has so far attempted to evaluate linguistic choices during hazard situations, we treat navigational behaviour as situational construals that are reflected in the visual and linguistic modalities of the dataset [48–51]:

**Hypothesis 1**. Navigational strategies of people posting images tagged with ‘nature’ and/or ‘landscape’ reflect the experiences of those who are not local to the area [52], i.e., visitors or tourists who like to spend time near watercourses or bird watching [53–55]. Therefore, when flood risks are communicated for a place with which they are not that familiar, they are not able to navigate these areas purposefully or to appreciate the dynamics of the flood event.

**Hypothesis 2**. The movement of people posting images with the tag ‘flood’ is different to that of the previous group. It is expected that such people are familiar with the area and what localised inundations can mean for the entire neighbourhoods [56], therefore they will demonstrate an orientation towards more structured mobility patterns in the course of the event (for example, after risk communication was put in place or during more severe stages of risk warnings).

**Hypothesis 3**. Similar to the temporary drift of lexical meaning [23, 53], navigational strategies reflected in images tagged with *alternative* lexemes, correspond to people’s experience of the landscape in its multiple states, from peaceful to the most dangerous [57] and, therefore, should demonstrate the most structured mobility patterns (i.e.,‘route-following)’ compared to the previous two categories.

### Datasets

#### YFCC100M

We used the Yahoo Flickr Creative Commons 100M (YFCC100M) dataset [58] containing a list of images and videos uploaded to the Yahoo! Flickr platform between April 2004 and August 2014. All the audio-visual material provided in this database is licensed under one of the Creative Commons copyright licenses (CC:BY).

#### Flood stages and risk communication

Flood stages are used to describe the progress in covering the designated flood risk areas with water. The main principle behind the designation of flood risk areas is topographic gradient [59]. Originally derived from direct geodetic surveys, now floodplains are designated with the help of more dynamic remote sensing techniques, using repeat high resolution ortophotography and photogrammetry.

Designations of topographically defined flood risk areas are used for various purposes. For example, insurance companies use them to automatically identify at-risk properties. Also, depending on the flood stage progression, flood risk areas are used by the authoritative environmental bodies (like the Environment Agency in the UK) to inform the public and organise rescue and evacuation campaigns.

In the UK, there are three types of risk communication messages corresponding to the stages of event severity: Alerts (‘*Flooding is possible*. *Be prepared*’), which are used from two hours to two days in advance of flooding, Warnings (‘*Flooding is expected*. *Immediate action required*’), which are used from half an hour to one day in advance of flooding and Severe flood warnings (‘*Severe flooding*. *Danger to life*’), which are put in place when flooding poses a significant threat to life.

**Fig 1:**
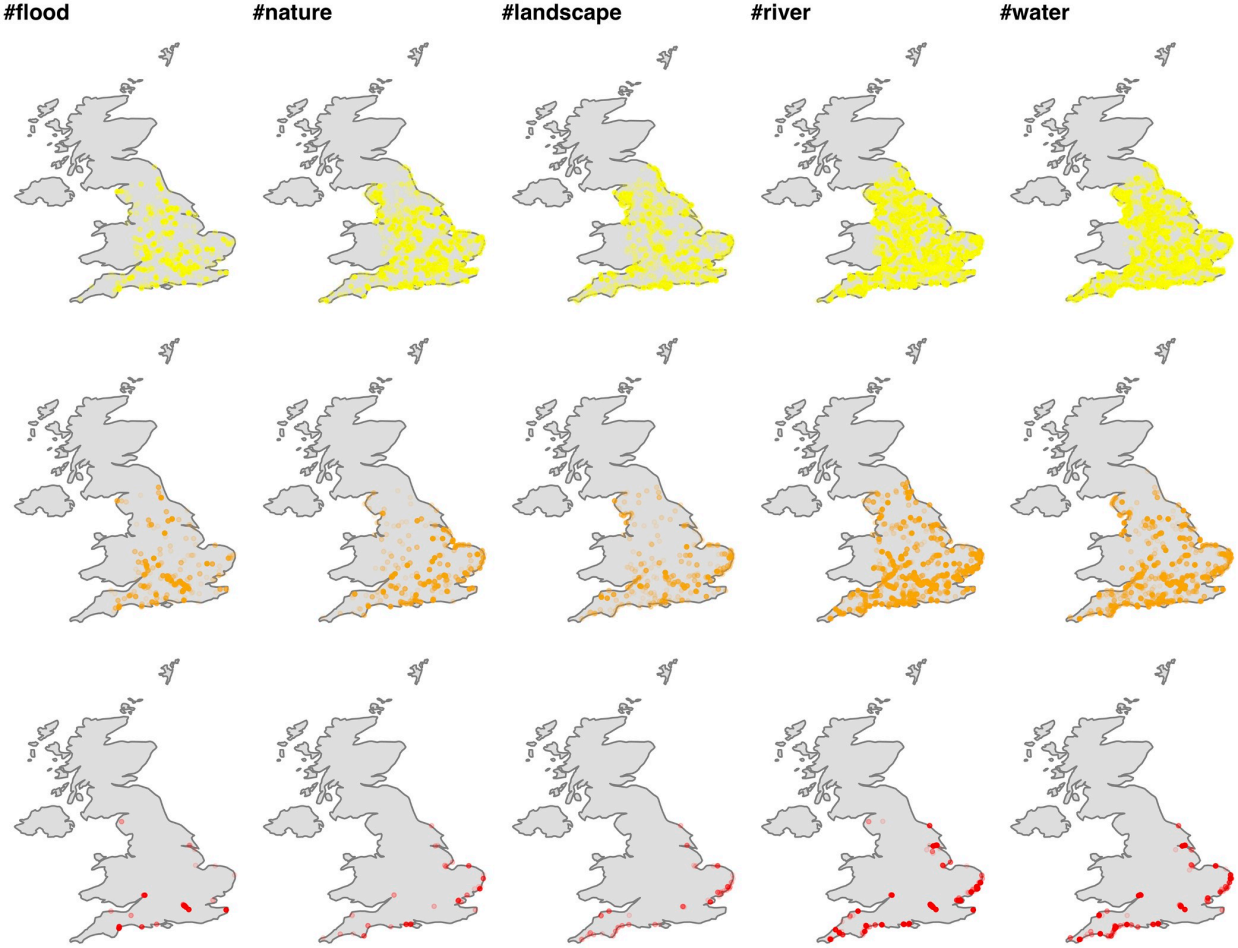
Spatial extraction of social media data. Distribution of geo-located Flickr tags uploaded to the platform during 2004-2014 within spatial designations used as communication units for *flood alert* (yellow), *warning* (orange) and *severe warning* (red) messages by the Environment Agency, UK.

Spatial designations of floodplains under Alert, Warning and Severe warning statuses and historic records of risk communication are available from the Government Data Portal (https://environment.data.gov.uk). The spatial intersection of these areas with the Yahoo Flickr posts is illustrated in Fig 1.

**Fig 2:**
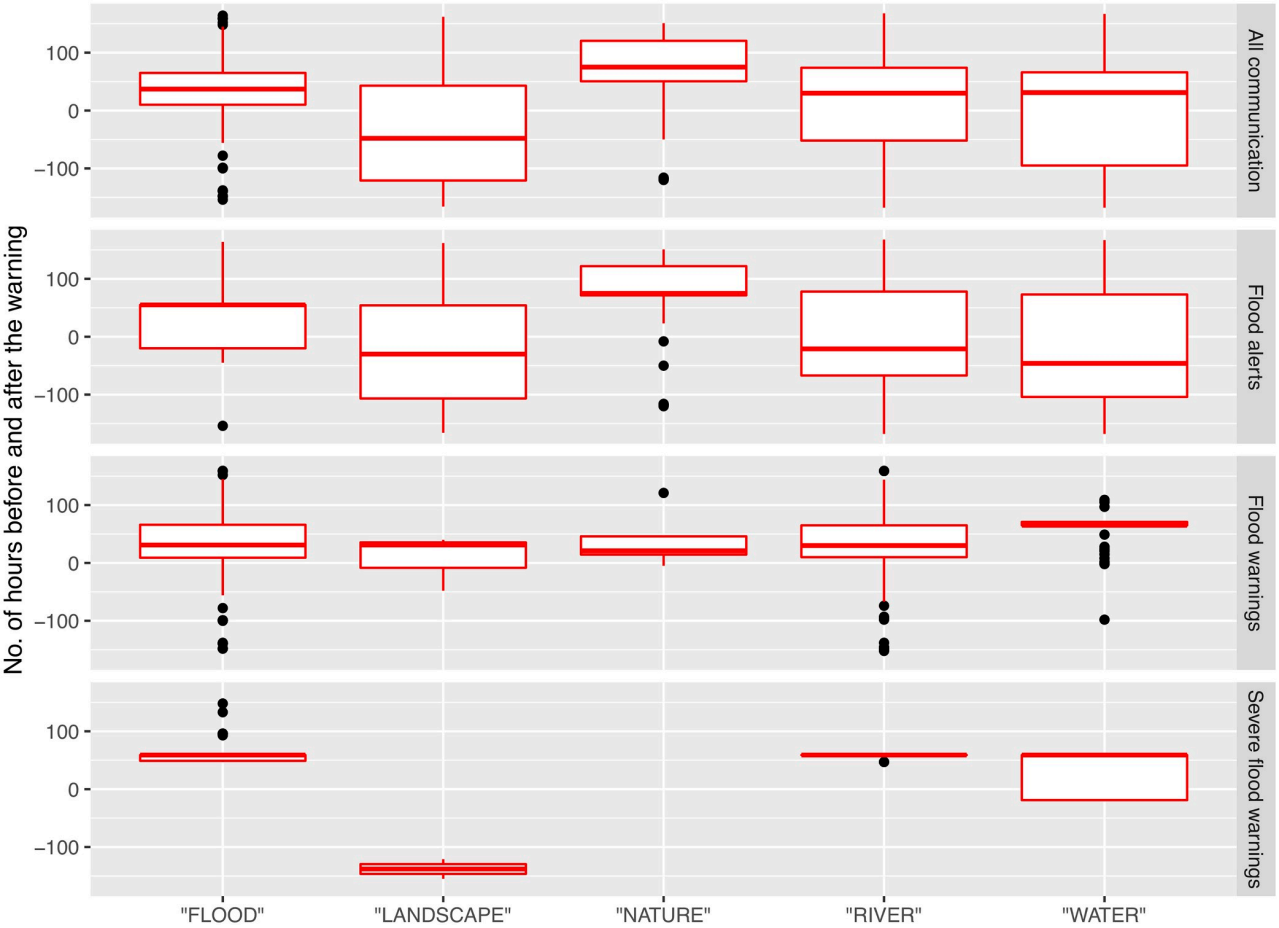
Temporal extraction of social media data. The temporal distribution (±100 hours) of tags around announced major flood events in the UK (2004-2014), using the 3-level flood risk communication system (alerts, warnings and severe warnings).

For designations of the ‘before’ and ‘after’ periods around flood risk communication, we selected 100 hours (approximately four days) in each direction around the timing of the announced risk status for each designated floodplain. The temporal distribution of relevant tags around ensembles of flood events 2004-2014 is illustrated below (Fig 2).

### Methods

There are two types of information we needed to extract from the social media dataset: **(a)** classification of scenes into categories of ‘objects’ and ‘landmarks’ posted before and after flood risk warning messages across all three stages of event severity (alerts, warnings and severe warnings), and **(b)** semantic relatedness of identified ‘objects’ and ‘landmarks’. This information was then subsequently related to the two types of navigation behaviours, ‘route-following’ and ‘wayfinding’, which can be used to characterise participating crowds as *locally experienced* or *naïve*, respectively.

In the case of the allocentric strategy (‘wayfinding’ behaviour) there are no obvious landmarks in sight, as well as no obvious connections between places. So, whilst adapting this statement to the properties of our data, it can be argued that *landmark-equivalent* corresponds to the *well-defined scene* associated with the highest probability value by the classification algorithm [42]. Connections (or their absence) between places can be also expressed with the help of the statistical probability of co-occurrences of scenic categories near each other, for example, in news outlets, which comprise a substantial topical corpus on natural disasters due to their ‘newsworthiness’ [60]. In the case of the egocentric strategy (‘route-following’ behaviour), the situation is opposite, where we should expect an increased number of well-defined, typical scenes with strong semantic connections.

#### ‘Deep’ image classification into ‘objects’ and ‘landmarks’

For natural scene classification we used the pre-trained Places CNN from MIT [61, 62], which classifies images into 365 scene categories. This dataset was designed to account for the human visual cognition system and is widely used for training classifiers to recognise high-level visual tasks, such as object detection, scene classification or event prediction. Each scene category is described with a two-tier labeling system, where simple nominal semantic categories (such as ‘road’ or ‘forest’) are associated with their functional counterparts (e.g., ‘broad leaved forest’, ‘mixed forest’, ‘city road’ or ‘desert road’). Following this classification, each image was allocated up to five scene categories and each of these values were used to make a decision whether the classified scenes corresponded to the categories of ‘objects’ or ‘landmarks’.

#### Jaccard distance

As our analysis was conducted across ensembles of spatial units (i.e., floodplains) and temporal segments (‘before’/‘after’ events) we used the metrics of the compositional dissimilarity across extracted spatio-temporal groups of images. For this purpose we chose *Jaccard distance* [63], which reflected the degree of dissimilarity between situational scene ensembles (binary comparisons between the lists of scenes A and B in Eq 1) and, in our case, aimed to test whether people tend to focus on the same of different areas during the various stages of flood events.

$$\begin{array}{c} d_{J}=\frac{\left|A\cup B\right|-\left|A\cap B\right|}{\left|A\cup B\right|} \end{array}$$ (1)

#### Semantic density of complete graphs

It can be argued that since environmental spaces require ‘panoramic’ observation to be effectively perceived [64, 65], the scenes-snapshots they are composed of also possess some kind of semantic interaction, due to crowds’ attention to, for example, important aspects of flood events (e.g., dramatic scenery of flooded houses and gardens, submerged vehicles, etc.). Therefore we can use *interactional* methods for their estimation, where nodes would correspond to the scene names and edges would reflect the strengths of their semantic similarities (i.e., weights). We therefore decided to turn to fundamental graph methods, which aim to explore semantic relatedness of scene clusters posted around each type of flood event (moderate (‘Alerts’), severe (‘Warnings’) and dangerous (‘Severe warnings’)), in order to visualise semantic pathways between previously identified ‘objects’ and ‘landmarks’. We therefore observe that the complete model of spatial navigational behavior for the area *A* during the time interval (*t*_1_ − *t*) resembles the shape of the weighted graph *G (E,V,w)*, where *w:E* → *eVal* and *eVal* represents the set of potential graph weights.

It can be argued that this type of situational *semanticity* can be analysed with the help of traditional embedding methods, where a model is usually powered by the domain-specific corpora and is used to extract semantic weights between lexical items (names of the scenes in our case) based on their co-occurrences. Following this principle, we used a standard *word2vec* cosine similarity algorithm for weights compilation, where semantic similarity between two lexical concepts *A* and *B* is represented as:

$$\begin{array}{c} \cos\left(\theta \right)=\frac{A\cdot B}{\left\|A\|\|B\right\|}=\frac{\sum_{i=1}^{n}A_{i}B_{i}}{\sqrt{\sum_{i=1}^{n}A_{i}^{2}}\sqrt{\sum_{i=1}^{n}B_{i}^{2}}} \end{array}$$ (2)

Our algorithm was based on the pre-trained Google word2vec model (https://code.google.com/archive/p/word2vec/) containing three million words and phrases, which has been trained on Google News data (around 100 billion words) and fitted using 300-dimensional word vectors (features).

Finally, we estimated sets of graph densities to be compared with each other using proportions between actual and potential semantic weights, where 0 means that scenes are semantically unrelated (conditions of poor or lack of orientation) and 1 illustrates topically connected clusters of the natural scenes:

$$\begin{array}{c} d_{G}\left(A,\Delta t\right)=\frac{\sum \left(w:E\right)}{\sum eVal}. \end{array}$$ (3)

## Results

### Compositional dissimilarity

**Fig 3:**
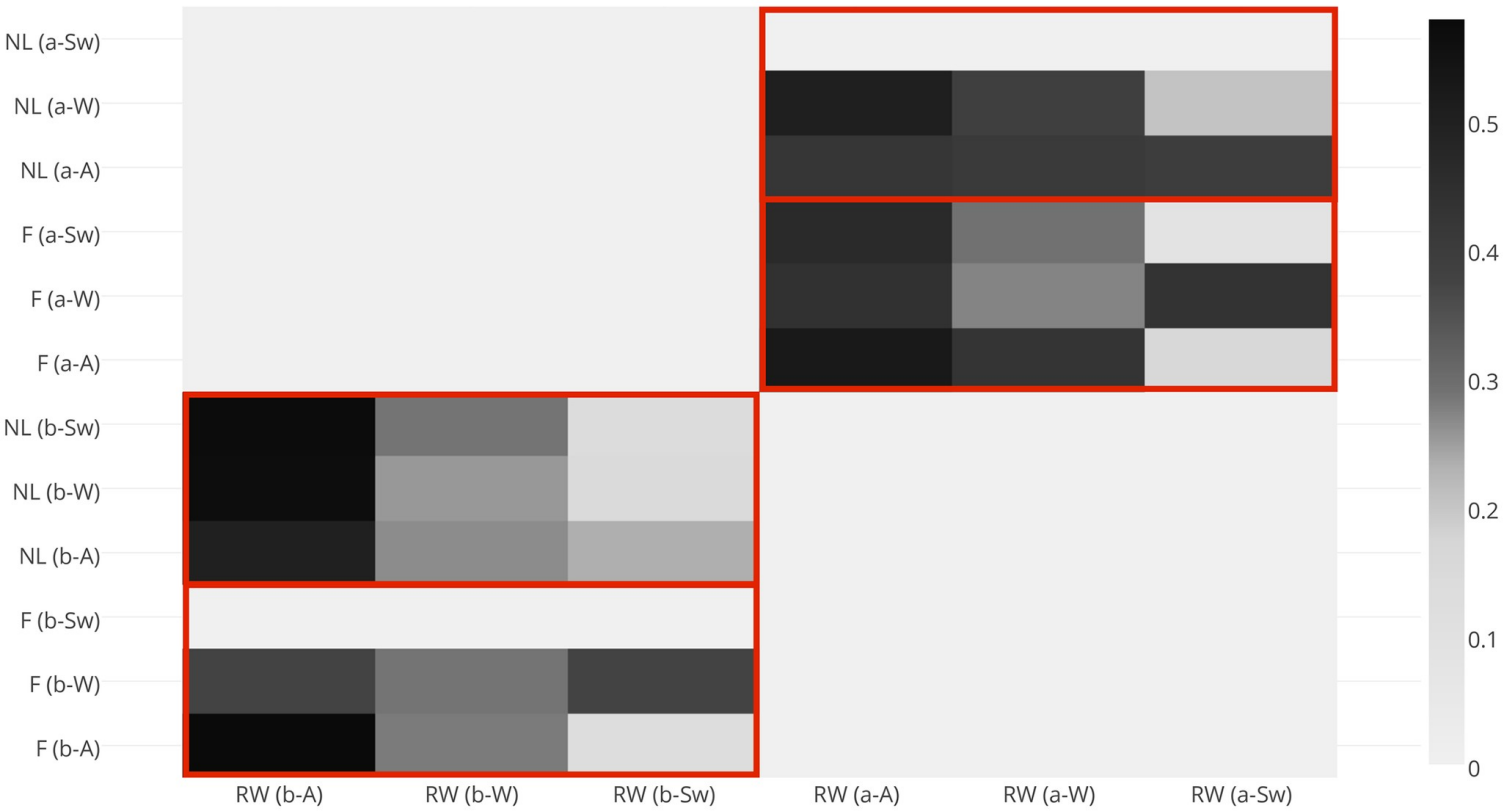
Jaccard distance between the scenes, tagged with neutral, positive and risk-signalling words posted before and after flood events 2004-2014. These results illustrate that the compositional distance between ‘neutrally’ tagged photographs and the two other sets generally decreases with event severity, both before and after risk communication messages. Zero values here correspond to ‘no data’. Abbreviations used: F(b-A; b-W; b-Sw): images tagged with words ‘flood’; RW(b-A; b-W; b-Sw): images tagged with words ‘river, water; NL(b-A; b-W; b-Sw): images tagged with words ‘nature, landscape’ and posted before authoritative flood alerts, warnings and severe warnings, respectively; F(RW, NL)(a-A; a-W; a-Sw): correspond to the sets of images, posted after authoritative flood risk communication.

First of all, we decided to look into how scenes tagged with alternative (textitneutral) lexemes (‘river’, ‘water’) differ from the two other groups of risk signalling (‘flood’) and benchmark words (‘nature’, ‘landscape’) (Fig 3).

**Fig 4:**
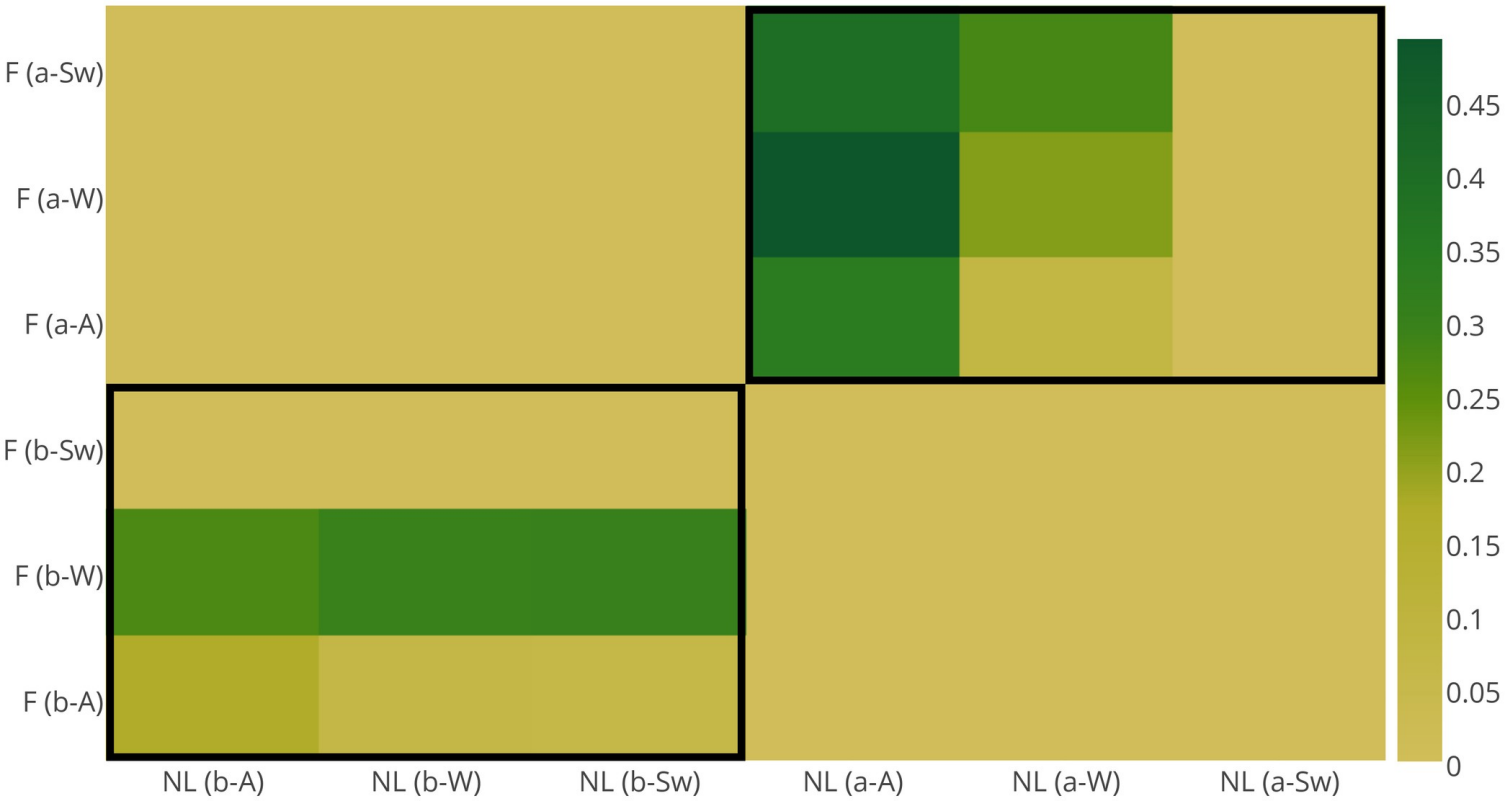
Jaccard distance between scenes tagged with positive (‘nature’, ‘landscape’) and risk-signalling (‘flood’) words posted before and after flood events 2004-2014. These results illustrate that before risk communication the dissimilarity increases evenly between ‘flood’-tagged and positively-tagged scenes with the increase of the event severity. After risk communication it also evenly decreases with event severity. This can be indicative of the fact that the perceived event severity affects segregation of the visual material in the same manner as authoritative risk communication, where the former segregates crowds according to the perceived danger, whilst the latter re-focuses their attention back onto familiar landscapes.

Here, neutral lexemes, which have previously demonstrated a transient shift of meaning around flood events [23], show an increased structural dissimilarity with both sets of words and this distance gradually decreases with the increase of event severity, for both cases before and after official risk communication messages. This can be indicative of the fact that during the early stages of flood events, lexemes that are prone to semantic drift under the influence of an approaching hazard are associated with different sets of scenes and, as the hazard evolves, the similarity between scenes increases. However, this step required subsequent comparison of scenes tagged with event descriptors (‘flood’) and positive words (‘nature’, ‘landscape’) (Fig 4).

Here we can observe that the compositional distance of positively tagged scenes posted before flood risk communication varies very little with event severity and this pattern is replicated for the ‘flood’-tagged scenes after authoritative warnings. This suggests that ‘flood’-tagged scenes hold the potential to discriminate between the severity of evolving flood events before risk communication, whilst positively tagged material have the potential to indicate post-event recovery when analysed alongside each other. However, definitive conclusions are difficult to draw because of the lack of ‘flood’-tagged material posted before severe warnings and positively-tagged scenes after.

**Fig 5:**
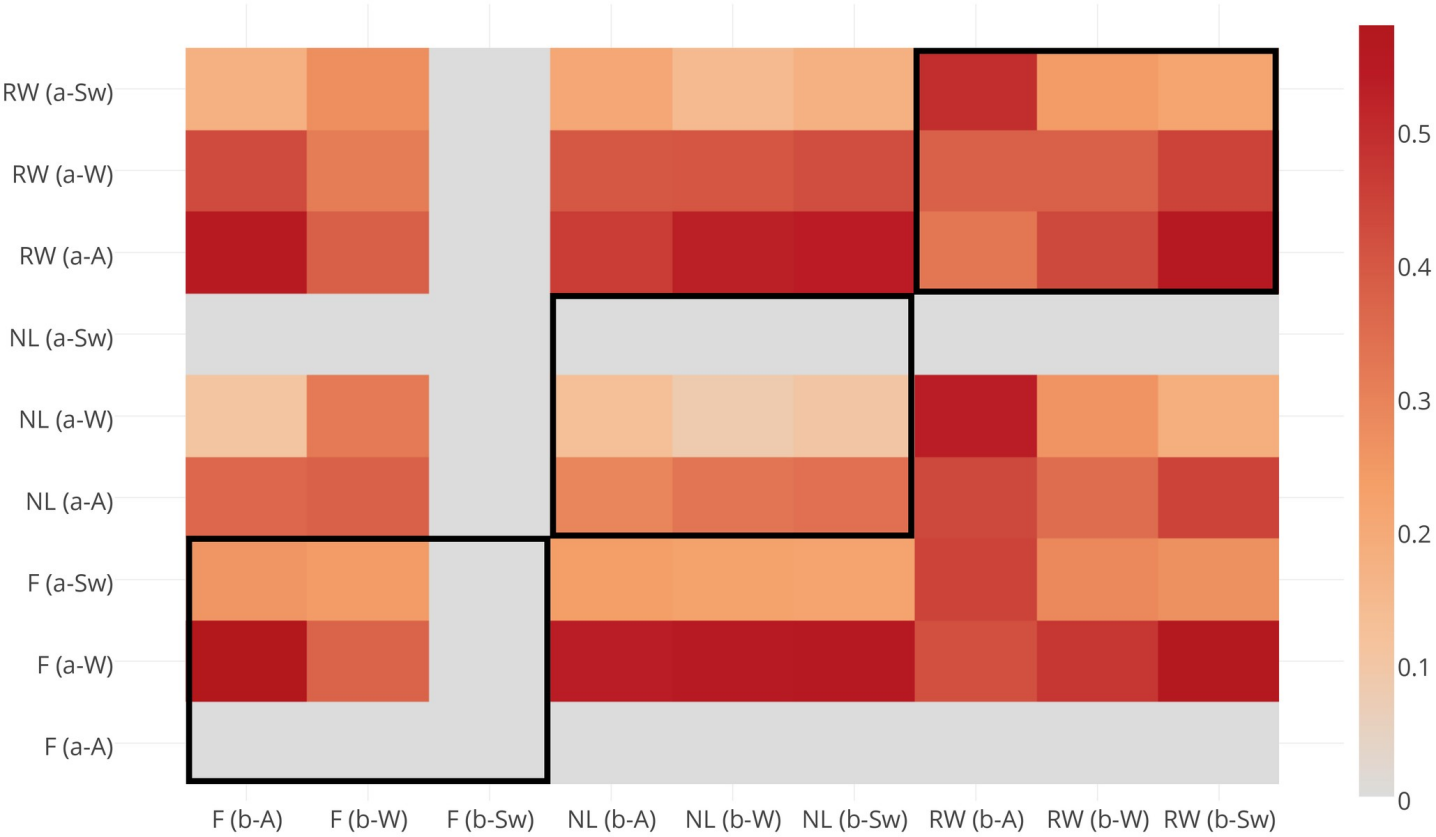
Compositional Jaccard distance between the sets of images posted *before* (horizontal axis) and *after* (vertical axis) ensembles of flood events 2004-2014. The results illustrate that scenes tagged with *alternative* lexeme-candidates for situational semantic shift demonstrate the highest compositional distance before and after flood risk communication, which is also independent of the event severity.

Finally, we looked at the compositional distance that the same three sets of lexemes tend to exhibit between themselves before and after authoritative risk communication (Fig 5). The results show the biggest structural distance in case of the *alternative* lexemes (‘river’, ‘water’), and the smallest for the case of positively-tagged scenes ‘nature’ and ‘landscape’, with risk-signalling material occupying a somewhat intermediate position between both groups.

Looking at these sets of results it is therefore possible to conclude that event-specific semantic drift of the neutral words (‘river’, ‘water’) discovered in our previous work [23] is also supported by the compositional dissimilarity of the images with which they are associated. Despite their temporal correlation with both sets of lexemes (positive and risk-signalling ones), the structural dissimilarity of their associated scenes across both sets—which decreases with event severity—may be indicative of the discriminatory potential for the severity of evolving hazards before authoritative risk communication takes place, as well as of varying (according to event severity) coping mechanisms of crowds after formal announcements of risk states. What it is important to find out, however, *is what kinds of crowds* tend to manifest their perceptions in such ways before and after official flood risk communication.

### Navigation strategies

#### Spatial focus

The results obtained so far generally aligned with findings confirming that asymmetrical lexical behaviour is more likely to be encountered in cases of unexpected events. However, since we are interested in crowd dynamics at the *sub-event* level, we need to look at how associated visual material is capable of differentiating types of exposed populations.

**Fig 6:**
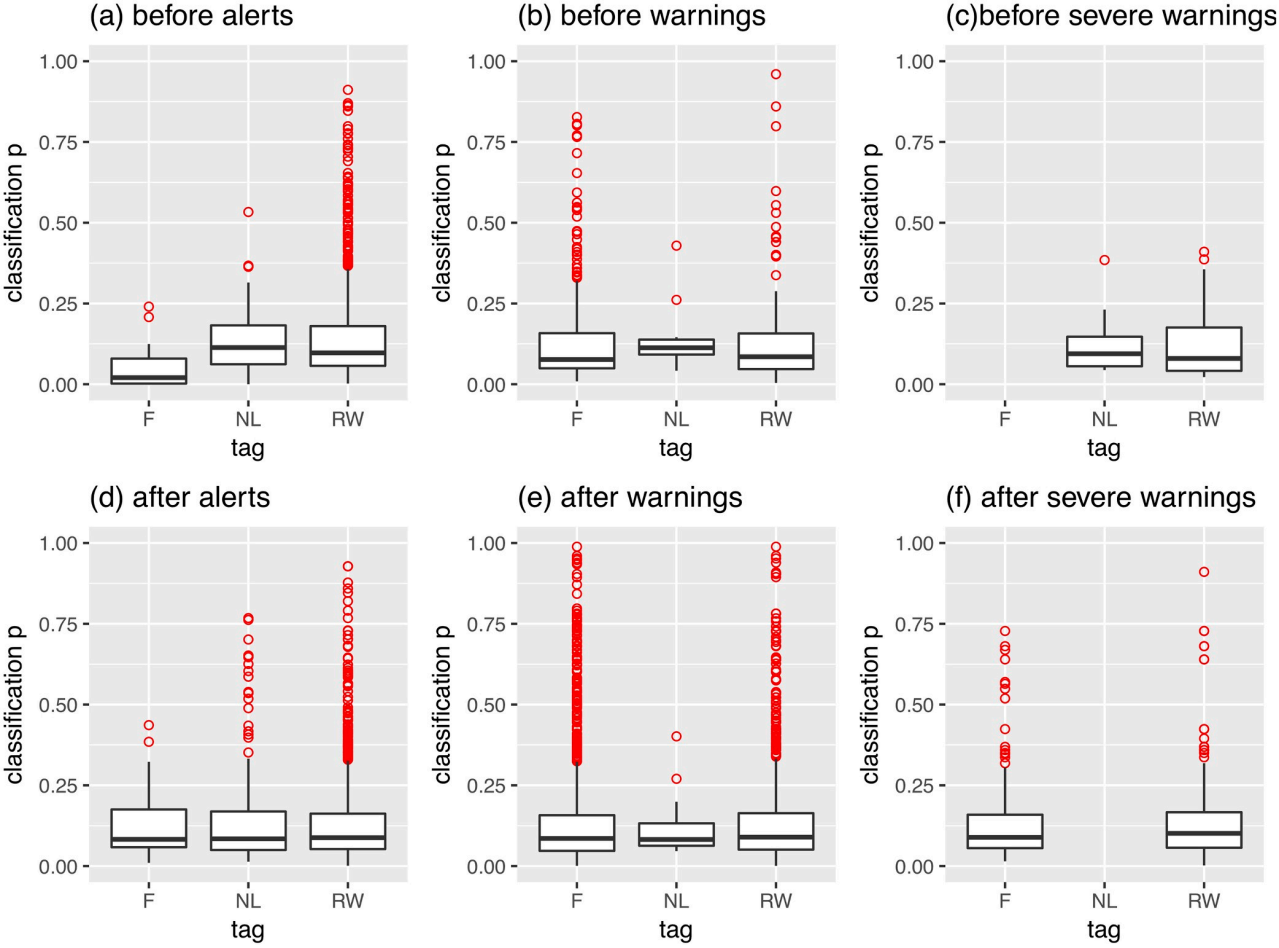
Distribution patterns of natural scene classification probabilities of the Flickr images posted around flood events in the UK (2004-2014).

Fig 6 illustrates results of the deep learning image classification with help of the pre-trained Places CNN (See Methods section above). Here we observe very little variation between the strengths of scene classification across all three groups of images associated with positive (‘nature’, ‘landscape’), negative (‘flood’) or neutral (‘river’, ‘water’) semantic tags. Since classification probability is associated here with one of the components of the spatial navigation modes (specifically *spatial focus*), we can therefore conclude that the bulk of images posted before and after flood risk communication reflects the *allocentric* (‘wayfinding’) type of crowd behaviour.

We also can observe the presence of one-sided outliers [66] representing groups of scenes with above average probabilities of class attribution (p>0.5). In our case, they are represented by groups of values significantly higher than Q3 (third quartiles) across all scenarios, thus rendering them ‘true’ outliers, which, according to the theory of spatial navigation in environmental spaces, have properties of ‘landmarks’ (i.e., highly focused, well-defined scenes). From the Fig 6 we may make the following observations:

1. Images associated with *alternative* tags ‘river’ and ‘water’ have the highest number of ‘true’ outliers, corresponding to *egocentric* navigational patterns (i.e., ‘route-following’ behaviours);
2. Across all tags, unlike *allocentric*, *egocentric* behaviours can better differentiate event severity than pre- and post-warning intervals of the same types of events. In case of the latter, we observe only a slight increase in the number of outliers, continuing patterns emerged before authoritative risk communication was put in place;
3. Previously found differences in structure, coupled with the lack of noticeable differences in the spatial focus of images posted before and after risk communication, suggest that event specific semantic drifts are products of the same crowds, which can change the ‘objects’ of their attention, but not their *focus* to evaluate them with (i.e., *familiarity* with the local environment or lack thereof);
4. In conditions of quickly changing landscapes at the beginning of flooding hazards, the majority of scenes are representative of ‘wayfinding’ behaviour, however, a substantial fraction of images tagged with ‘river’ and ‘water’, also represents more focused ‘landmarks’ and is indicative of ‘route-following’ crowd behaviours. These behaviours are then subsequently picked up by the images tagged with ‘flood’-related words.

#### Spatial orientation

**Fig 7:**
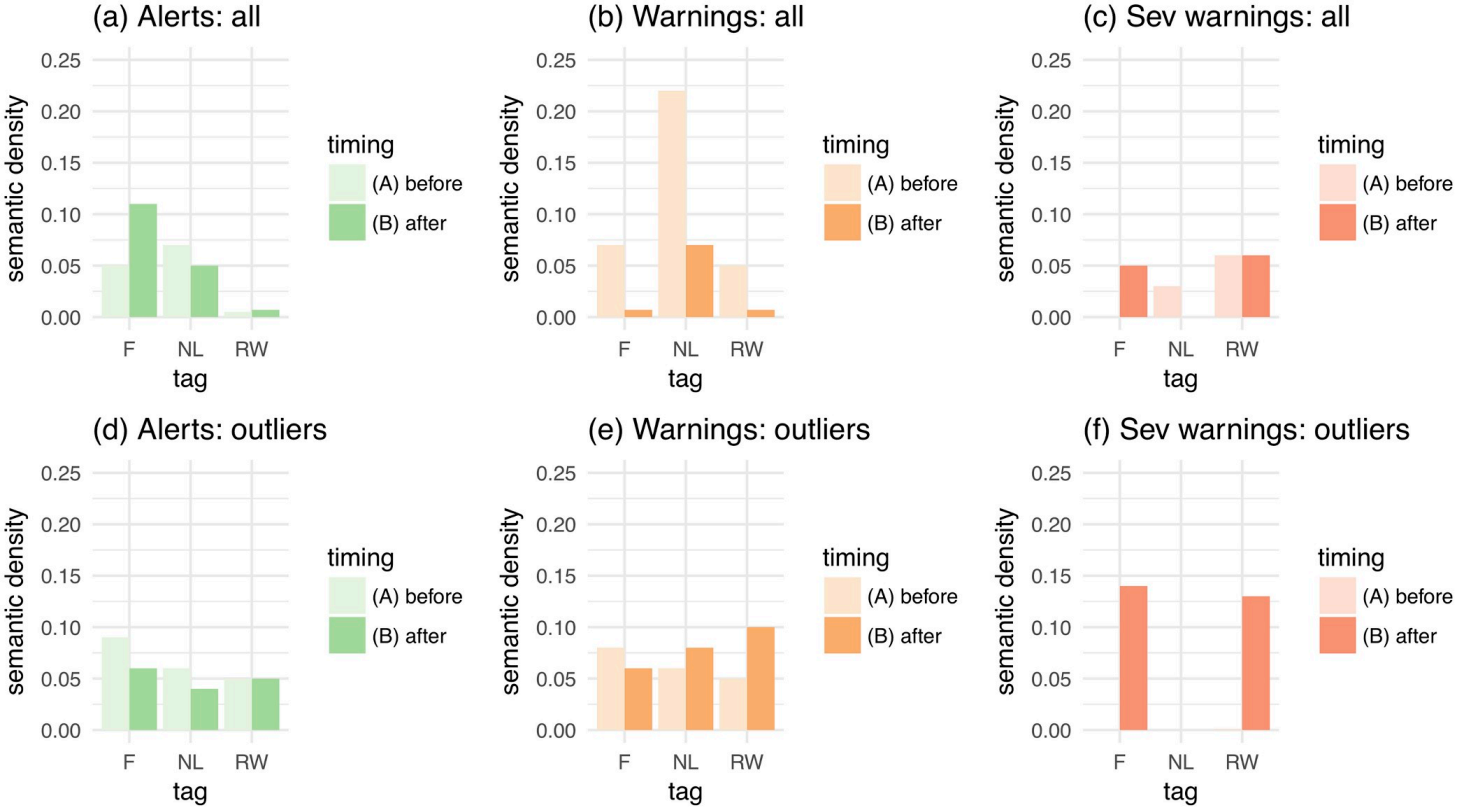
Semantic density of navigational frames captured by images on the Yahoo! Flickr platform posted before and after official flood risk communication.

In order to get a complete picture of the types of spatial navigational behaviour, we also need to take a look at the values of *semantic density* between scene clusters, which are indicative of coherent spatial orientation. Fig 7 illustrates the following findings:

1. The semantic density of all ‘flood’-tagged scenes gradually decreases with the increase in event severity. Following previous sets of findings, this phenomenon is coupled with the simultaneous increase of spatial focus. This means that behaviour here is becoming *predominantly egocentric* (i.e., ‘route-following’) as a hazard gets more severe. In the case of semantically unstable material the trend is exactly the opposite: increased semantic density is accompanied by focus decrease, thus leading to a *predominantly allocentric* (‘route-following’) behaviour;
2. Amongst groups of outliers and as compared to the entire datasets, the most dramatic examples of semantic density are for scenes tagged with semantically unstable words and this density also increases with event severity. It is therefore possible to conclude that after emergence of *egocentrically*-orientated ‘flood’-tagged scenes, the rest of the *alternative* lexemes start losing their significance as *risk-signallers* and prepare to *mutate* back to more positive connotations (i.e., ‘nature’ and ‘landscape’).

## Discussion

Our results have demonstrated that different types of crowdsourced, lexical material with associated visual media related to the topic of environmental perception of risk have the potential to not only sense an approaching flooding hazard, but also get insights into its stages, i.e., from the least to the most severe.

Our findings also point to the fact that it is important to consider the interplay of various ontologically connected groups of keywords in order to be able to uncover fully event dynamics with the help of multi-modal social media. According to our results, the bulk of spatial navigational patterns across all types of scenes is represented by ‘wayfinding’ navigational strategies. However, they significantly differ in the structure of their outliers, where natural scenes associated with semantically unstable material (‘river’, ‘water’) demonstrate the earliest crowd segregation into ‘wayfinders’ and ‘route-followers’, a trend, which is then subsequently picked up by the actual ‘route-demonstrating’ material tagged by the word ‘flood’. Therefore, we can conclude that the second hypothesis can be accepted. However, the third one is only partially true as, instead of demonstrating the most structured mobility patterns, the group of scenes tagged with *alternative* lexemes only indicates the strongest *tendency* towards crowds with *egocentric* characteristics. Similar to the second hypothesis, the first is also accepted as, generally, the navigational strategies of crowds posting material tagged with ‘nature’ and/or ‘landscape’ reflect the experiences of people who are not local to the area, which is supported by the bulk of the ‘wayfinding’ navigational patterns implied from corresponding photographs.

## Conclusions

Our findings suggest that semantically unstable, lexical material in posts on social media can be used by different types of event participants. This divergence of meaning may lead to the emergence of ‘route-following’ scene ensembles tagged with direct event descriptors ‘flood’, hence providing local navigational knowledge *before* official flood risk communication takes place. *After* risk of flooding is announced these posts start acquiring a structural resemblance to scenes tagged with ‘nature’ and ‘landscape’, which is the strongest in cases of more severe events, thus indicating an end of the *proactive crowd sensing* stage and the beginning of more guided, *passive* attitudes towards hazard events. These are marked, however, by much a stronger visual focus (i.e., *observational* approach) than positively-tagged scenes.

The importance of this analysis lies in the fact that making use of social media can help us to expand substantially operational knowledge regarding the locations of the most vulnerable populations during hazardous events, as well as to make use of valuable local knowledge of how to efficiently manoeuvre using local landmarks and their semantic connectivity. These strategies generally align with risk perception studies, highlighting the importance of social insights for designing and evaluating risk communication programs.

## Limitations and future work

Although the choice of social media data to study human perceptions of natural hazards was a straightforward one, there is also a number of limitations associated with the choice of data and subsequent research design.

First of all, the mere fact that our data derived from the social media platform suggests that the data coverage will be uneven, and as a consequence this can find reflection in the spatial component of our multi-modal dataset, since people living or visiting flood risk areas are not necessarily representative of the demographics of social media platform as a data provider. Second, data analysis on the archival data does not allow us to extend our methodology towards real-time risk signalling, which limits applicability of this study to its mere validating/exploratory role.

Also, although this analysis is indicative of the discriminatory potential for the severity of evolving hazards before authoritative risk communication takes place, it can be advised that similar studies would significantly benefit from testing across much wider range of the hazard events or risk-related situations before the definite conclusions about the full potential of semantically drifted material for event segmentation on social media can be made.

In addition, it is advisable that any results derived from the social media data as immersive source of large scale information are subsequently empirically validated with participatory studies, designed for/across range of representative geographies or spatial scales.
